# Prevalence of celiac artery compression by median arcuate ligament in patients with splanchnic artery aneurysms/pseudoaneurysms submitted to endovascular embolization

**DOI:** 10.1007/s00261-023-03844-x

**Published:** 2023-02-18

**Authors:** Ana Paula Borges, Célia Antunes, Paulo Donato

**Affiliations:** 1 Medical Imaging Department, Coimbra University Hospitals, Coimbra, Portugal; 2 Faculty of Medicine, University of Coimbra, Coimbra, Portugal; 3 Academic and Clinical Centre of Coimbra, Coimbra, Portugal

**Keywords:** Median arcuate ligament, Celiac artery compression, Splanchnic circulation, Aneurysm, False aneurysm, Endovascular procedure

## Abstract

**Purpose:**

To study the association between median arcuate ligament compression (MALC) of celiac artery (CA) and splanchnic artery aneurysms/pseudoaneurysms (SAAPs) submitted to endovascular embolization.

**Methods:**

Single center retrospective study of embolized SAAPs between 2010 and 2021, to evaluate the prevalence of MALC, and compare demographic data and clinical outcomes between patients with and without MALC. As a secondary objective, patient characteristics and outcomes were compared between patients with different causes of CA stenosis.

**Results:**

MALC was found in 12.3% of 57 patients. SAAPs were more prevalent in the pancreaticoduodenal arcades (PDAs) in patients with MALC, compared to those without MALC (57.1% vs. 10%, *P* = .009). Patients with MALC had a greater proportion of aneurysms (71.4% vs. 24%, *P* = .020), as opposed to pseudoaneurysms. Rupture was the main indication for embolization in both groups (71.4% and 54% of patients with and without MALC, respectively). Embolization was successful in most cases (85.7% and 90%), with 5 immediate (28.6% and 6%) and 14 non-immediate (28.6% and 24%) post-procedure complications. Thirty and 90-day mortality rate were 0% in patients with MALC, and 14% and 24% in patients without MALC. Atherosclerosis was the only other cause of CA stenosis, in 3 cases.

**Conclusions:**

In patients with SAAPs submitted to endovascular embolization, the prevalence of CA compression by MAL is not uncommon. The most frequent location for aneurysms in patients with MALC is in the PDAs. Endovascular management of SAAPs is very effective in patients with MALC, with low complications, even in ruptured aneurysms.

**Graphical Abstract:**

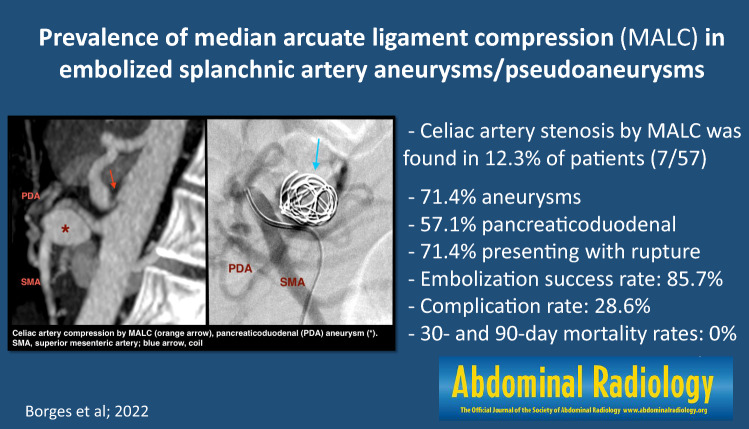

## Introduction

Median arcuate ligament (MAL) is a fibrous arch that joins the right and left crura of the diaphragm on either side of the aortic hiatus, usually at the level of the 12th thoracic vertebra, crossing the aorta superior to the celiac artery (CA) origin [1].

Celiac artery compression by MAL is increasingly being detected due to the widespread use of imaging studies [2]. In fact, it is suggested in the literature that in up to a third of people, MAL has a lower insertion and may compress the proximal portion of the CA, which is usually more severe in expiration, given the cranial displacement of the aortic branches [3]. It is considered to constitute one of the main causes of CA stenosis [4]. Still, it is mostly asymptomatic [2].

When this anatomy is associated with symptoms, it is named median arcuate ligament syndrome (MALS), whose prevalence is extremely low (0.002% of the population), and more frequently detected in female patients. Clinical presentation is variable and includes weight loss, postprandial epigastric pain, nausea, and vomiting, most frequently manifesting at the ages of 20 to 50 years [1, 2]. The diagnosis is usually only established after the exclusion of other more common causes of symptoms [5].

Two main mechanisms have been attributed to MALS, namely compression of vascular and nervous structures. Vascular compression alone does not seem to be enough explanation, given that adequate blood supply to the digestive organs is provided by the abundant collateral circulation via the mesenteric arteries. As so, it is hypothesized that the myoelectric activity of the smooth muscles may also be altered by the compression of the nervous splanchnic plexus fibers in the digestive tract. Either splanchnic vasoconstriction or direct stimulation of pain neurons may result in neurogenic-related pain [1].

On computed tomography angiography (CTA), the diagnosis is confirmed by the visualization of a focal narrowing at the origin of the CA with a hooked appearance, better depicted at arterial phase imaging in the sagittal plane [6].

The coexistence between CA stenosis or occlusion and aneurysms in its visceral arterial collaterals was first described in 1973 by Sutton and Lawton [1, 7]. Since then, several studies have reported an association between MALS and pancreaticoduodenal arcades (PDAs) aneurysm formation [2]. Hypothesized explanations for this phenomenon include the increased blood flow and hypertension in the small collateral arteries connecting the CA and the superior mesenteric artery (SMA) beds (enlarging them and eventually weakening its wall), and the increased arterial wall shear stress [1, 2, 8]. Besides, the dynamic nature of the CA compression may result in alternating anterograde and retrograde blood flow in these collaterals, with consequent turbulent flow [3]. Pancreaticoduodenal arcades aneurysms not related to MALS are mostly secondary to atherosclerosis in CA and its branches [9].

Endovascular treatment with CA angioplasty and stent placement may increase the blood flow to the CA, but surgical MAL incision (either laparoscopically or by open surgery) should be the standard therapy for MALS, given that the cause of the stenosis is the extrinsic compression by this ligament [1, 2, 10].

Despite the reported cases of PDAs aneurysm regression or stability following CA decompression in patients with MALS, the treatment of these aneurysms is recommended regardless of their size, given their significant risk of rupture, which is associated with a relatively high mortality rate. When possible, endovascular embolization (often with metallic coils) is currently considered the preferred option for both ruptured and unruptured aneurysms, given its less invasiveness and increased safety in recent years [1, 2, 6, 11]. Surgery is recommended in unstable patients and cases of endovascular treatment failure [1]. It is technically feasible but associated with a mortality rate of up to 30% in ruptured aneurysms [11]. A potential complication of PDAs aneurysm embolization in patients with CA stenosis is the ischemic damage to organs such as the liver or the pancreas, due to interruption of the collateral circulation [1].

However, being a rare disease, no therapeutic guidelines have been established for patients with splanchnic artery aneurysms and MALS. Celiac artery decompression to correct the hemodynamic imbalance in the PDAs is often recommended to prevent ischemia and aneurysm recurrence [1, 2, 10, 12–18], whereas others reported favorable outcomes with aneurysm embolization alone [1, 16, 19–27].

The main goal of this article was to determine the prevalence of median arcuate ligament compression (MALC) of celiac artery in patients with splanchnic artery aneurysms/pseudoaneuryms (SAAPs) submitted to transcatheter arterial embolization, and compare demographic data and clinical outcomes between patients with and without MALC. As a secondary objective, we analyzed other causes of CA stenosis and compared patient characteristics and outcomes between patients with different causes of CA stenosis.

## Material and methods

### Study sample

After institutional review board approval, we performed a retrospective review of adult patients submitted to endovascular embolization of SAAPs at the Interventional Radiology department of a tertiary-care institution, between January 1^st^ of 2010 and December 31^st^ of 2021, to determine the prevalence of MALC and compare demographic data and clinical outcomes between patients with and without MALC. As a secondary objective, we also compared patient characteristics and outcomes between patients with different causes of CA stenosis.

From the electronic medical records, we collected and reviewed information regarding patient demographics (sex, age, comorbidities including high blood pressure and diabetes mellitus, smoking habits, and alcohol consumption), clinical characteristics (clinical presentation and indication for embolization), and clinical outcomes (embolization success, immediate and non-immediate post-procedure complications defined by the occurrence during or in the same day of the procedure, or after that period, respectively, and mortality rates at 30 and 90 days).

Patients from other institutions admitted only for the procedure and discharged to another hospital were excluded from this analysis, due to lack of enough clinical and imaging data.

### CTA evaluation of celiac artery stenosis

The presence or absence of CA stenosis or occlusion was noted by means of analysis of CTA images (current, prior, or following embolization) and its cause was registered.

**Fig. 1:**
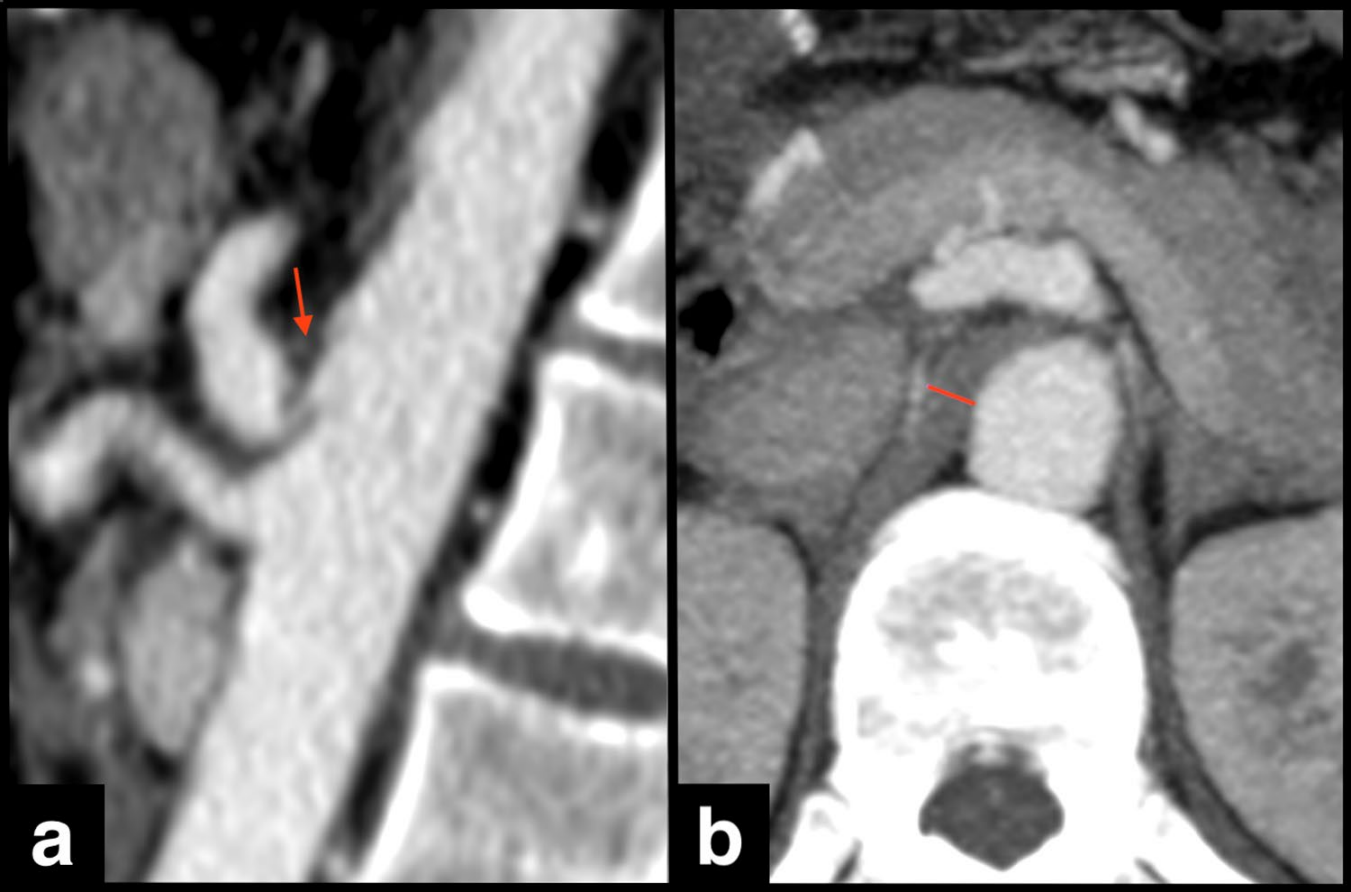
Celiac artery compression by median arcuate ligament. Sagittal (**a**) and axial (**b**) CTA images showing the classic hook-shaped configuration (arrow in A) with severe stenosis at the origin of the celiac artery (CA) and post-stenotic dilatation, in the absence of atherosclerotic plaques. The maximum thickness of median arcuate ligament was measured in the axial plane, at the level of the CA origin (**b**)

The CTA criteria used for diagnosing MALC included proximal CA obstruction or stenosis ≥ 50% with a hooked or U-shaped configuration, caused by a superior indentation by the thickened MAL seen on the sagittal plane, in association with a post-stenotic dilatation (Fig. 1a). The maximum thickness of MAL was measured in the axial plane, at the level of the CA origin (Fig. 1b). Atherosclerotic etiology was considered in the presence of CA obstruction or stenosis ≥ 50% caused by atherosclerotic plaques in its ostium, without the hooked or U-shaped appearance.

Features evaluated included the grade of stenosis (ratio between the greatest diameter of the post-stenotic dilated segment and the diameter of the most narrowed segment), length of stenosis, and the greatest caliber of the SMA, gastroduodenal artery (GDA), PDAs, and largest arterial branch in the body/tail of pancreas, measured in the arterial phase images.

### Definition and characterization of SAAPs

Aneurysms and pseudoaneurysms were distinguished based on their cause and associated findings. Aneurysms were considered in conditions of arterial wall weakening such as arteriosclerosis, fibromuscular dysplasia, medial degeneration, arteritis, and connective tissue diseases, as well as in portal hypertension and cases of unknown cause. Pseudoaneurysms were assumed in cases of inflammatory/infectious conditions, abdominal trauma, and post-surgical or percutaneous biliary interventions. Both were defined as a localized dilatation (> 1.5 times the expected normal diameter) of an artery [28].

Aneurysms were characterized either by conventional angiography or CTA (4-, 16- and 64-slice multidetector), when available, namely in terms of their location, size, morphology (i.e., fusiform or saccular), and presence of wall calcification and thrombosis. In patients with more than one, only the largest was considered.

### Embolization procedure

The embolization procedure was performed by one or more experienced interventional radiologists (> 10 years of experience). Anemia and abnormal coagulation markers motivated corrective measures before the intervention, namely if hemoglobin was < 8 g/dl, and the platelet count was < 20 × 109/L or INR > 1.5, respectively.

After puncture of the right common femoral artery under local anesthesia, a 4- or 5-F vascular sheath was placed. Subsequent cannulation was performed using a 4- or 5-F catheter of various shapes (shepherd hook, cobra, twist, Simmons). Angiographic images of the CA and SMA were obtained. Selective catheterization of the feeding arteries and SAAPs was performed with a coaxial microcatheter for transarterial embolization, mostly with microcoils and coils.

Technical success was considered if the aneurysm was excluded and hemorrhage ceased in cases of rupture.

### Statistical analysis

The statistical analysis was performed using IBM SPSS Statistics, version 28.0.1.1 (IBM Corp., Armonk, N.Y., USA). Categorical values were compared using Fisher’s exact test or chi-square test, as indicated. Student’s t-test or Mann–Whitney U test were used to compare continuous variables, as indicated. For comparative analysis, categorical variables with more than 2 categories were encoded in indicator variables. A *P*-value of < 0.05 was considered statistically significant.

## Results

**Fig. 2:**
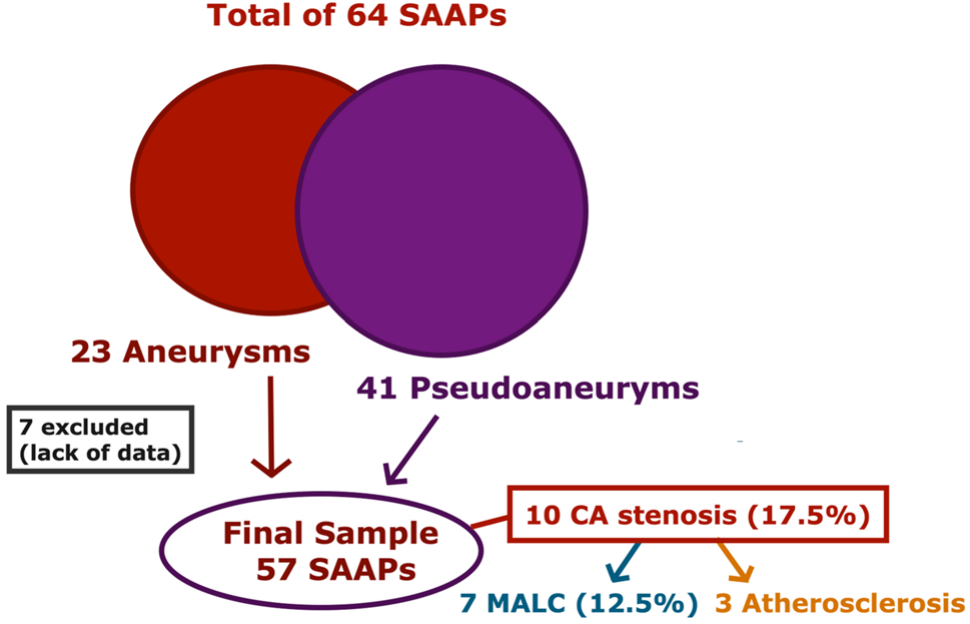
Schematic representation of patient selection and celiac artery (CA) stenosis prevalence and causes. MALC, median arcuate ligament compression; SAAPs, splanchnic artery aneurysms/pseudoaneuryms

During the study period, we found a prevalence of CA compression by MAL in 7 out of 57 patients submitted to endovascular embolization of SAAPs at our institution (12.3%), after the exclusion of 7 patients, due to lack of enough clinical and imaging data. Celiac artery stenosis was found in 3 other patients, caused by atherosclerotic plaques (Fig. 2). Minimal atherosclerotic plaques were found in the CA of three patients with MALC, but they were not occlusive.

**Fig. 3:**
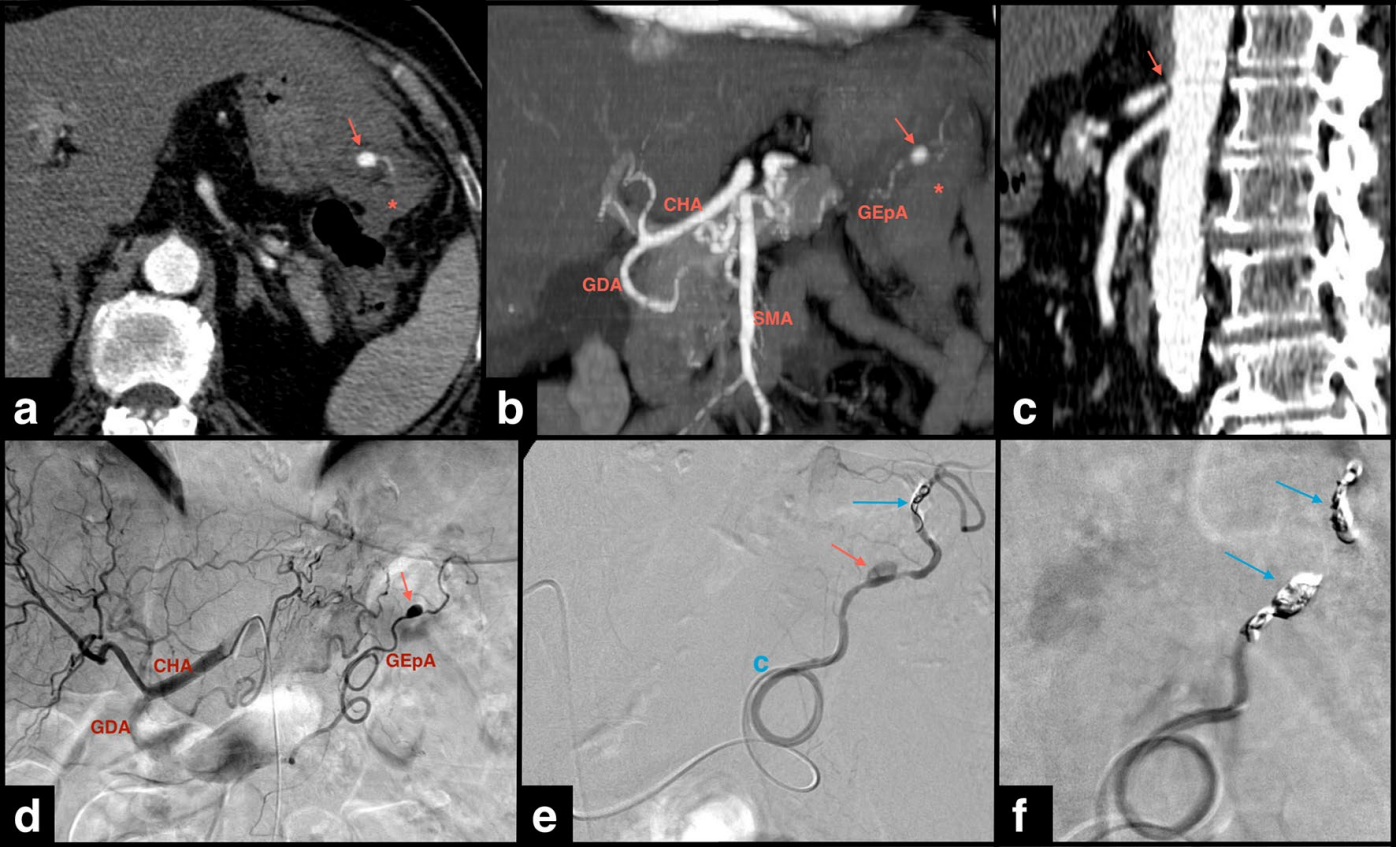
Ruptured aneurysm of the left gastroepiploic artery (GEpA) in a patient with celiac artery stenosis. Axial (**a**) and coronal MIP reconstruction (**b**) CTA images show the opacified aneurysm (arrows in **a**, **b**) with associated hemorrhage (*). Sagittal CTA image (**c**) shows the celiac artery compression by the median arcuate ligament (arrow). Arterial angiography images (**d**-**f**) show the GEpA aneurysm (red arrows). Both artery and aneurysm were embolized with microcoils (blue arrows), after selective catheterization with a coaxial microcatheter (**c** in image **e**). Post-embolization angiogram (**f**) shows no residual aneurysm opacification or bleeding. CHA, common hepatic artery; CTA, computed tomography angiography; GDA, gastroduodenal artery; MIP, maximum intensity projection; SMA, superior mesenteric artery

**Fig. 4:**
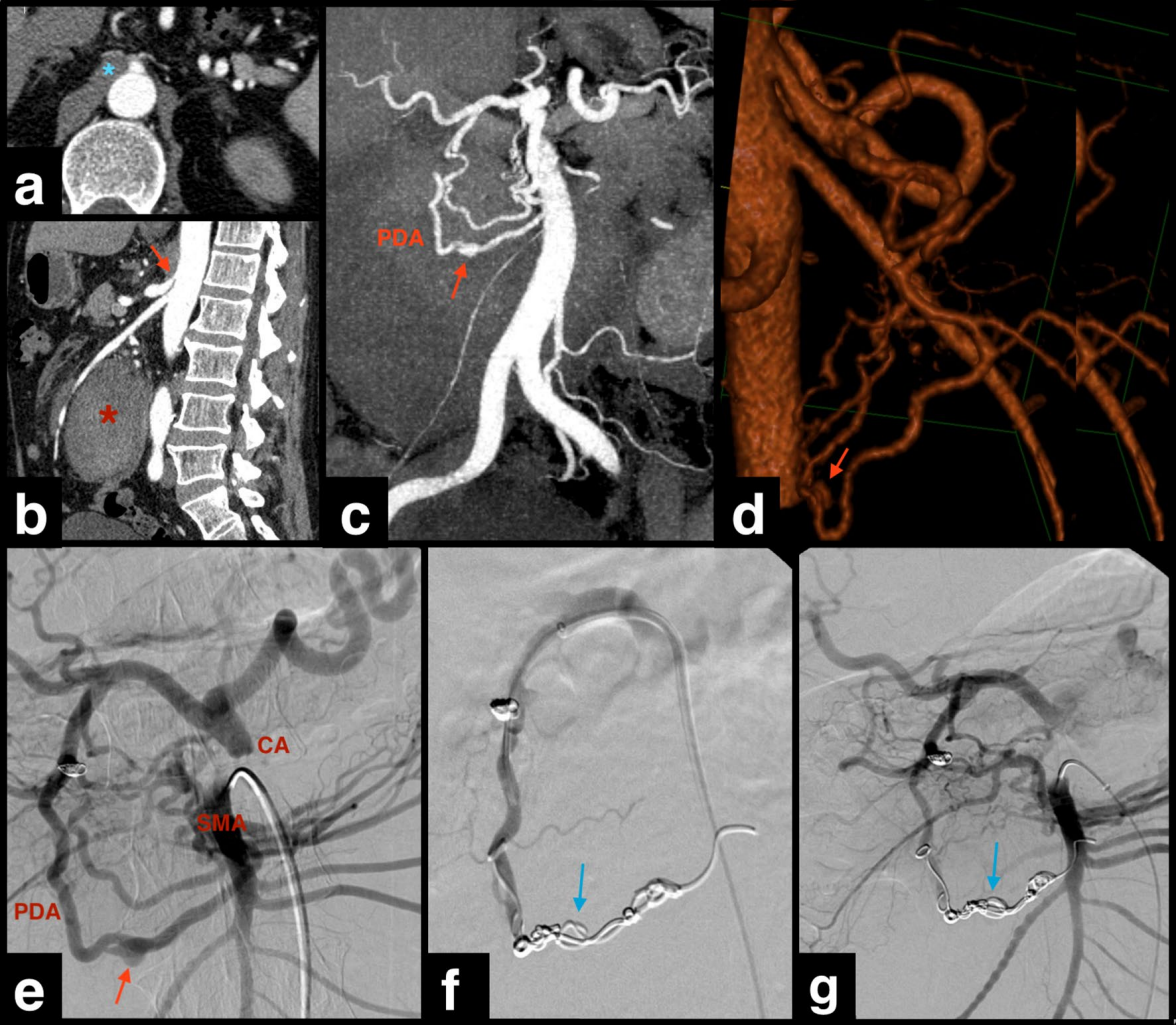
Ruptured aneurysm of the anterior inferior pancreaticoduodenal artery (PDA) in a patient with celiac artery stenosis. Axial (**a**) and sagittal (**b**) CTA images show the thickened median arcuate ligament (* in **a**), celiac artery compression (arrow), and a blood collection (* in **b**) secondary to rupture of a PDA aneurysm (arrow in **c**, coronal MIP reconstruction, and **d**, 3D reconstruction). Selective superior mesenteric artery (SMA) angiogram (**e**) shows severe celiac artery (CA) stenosis and the PDA aneurysm (red arrow). After selective catheterization (**f**), both artery and aneurysm were embolized with microcoils (blue arrows). Post-embolization angiogram (**g**) shows no residual aneurysm opacification. A “string-of-beads” appearance was noted in the ileocolic artery, indicative of segmental arterial mediolysis or fibromuscular dysplasia (not confirmed), suggesting an exacerbating and not only causative role of MALC in aneurysm formation and rupture. CTA, computed tomography angiography; MIP, maximum intensity projection; SMA, superior mesenteric artery

Statistical comparison between patients with and without MALC is shown in Table 1. The mean age of patients with and without MALC was 59.7 years (± 18.4) and 61.6 years (± 15.2), respectively (*P* = 0.763). The male gender was predominant in both groups (57.1% and 68%, *P* = 0.675). High blood pressure (HBP) and diabetes mellitus (DM) were present in 42.9% and 71.4% of patients with MALC, respectively, with lower prevalence in patients without MALC (32% and 30%, *P* = 0.675 and 0.084). Smoking and alcohol consumption were not clarified in more than three thirds of patients. The main indication for embolization was rupture with active bleeding in both groups, namely 71.4% and 54% of patients with and without MALC (*P* = 0.450), respectively (Figs. 3 and 4). Notably, a “string-of-beads” appearance was noted in the ileocolic artery of a patient with MALC and PDA aneurysm rupture, indicative of segmental arterial mediolysis or fibromuscular dysplasia (not confirmed), suggesting an exacerbating and not only causative role of MALC.

**Table 1:** Statistical analysis of cases with and without median arcuate ligament compression (MALC) of the celiac artery (CA)

Variables	MALC (*n* = 7)	No MALC (*n* = 50)	*P-*value	OR [CI 95%]
Mean age (years ± SD)	59.7 ± 18.4	61.6 ± 15.2	0.763	
*Gender*				
Male Female	4 (57.1) 3 (42.9)	34 (68) 16 (32)	0.675	
*Comorbidities (Y/UK)*				
DM HBP	3 (42.9)/ 0 5 (71.4)/ 0	16 (32)/ 4 (8) 15 (30)/ 4 (8)	0.675 0.084	
Smoking habits (Y/UK)	1 (14.3)/ 5 (71.4)	6 (12)/ 37 (74)	1.000	
Alcohol consumption (Y/UK)	1 (14.3)/ 5(71.4)	15 (30)/ 32 (64)	0.660	
*Clinical presentation*				
Asymptomatic Abdominal pain Intra-abdominal hemorrhage Upper GI bleeding Anemia Fever Hemobilia Nausea + Vomit Syncope	2 (28.6) 2 (28.6) 2 (28.6) 1 (14.3) 1 (14.3) 0 0 0 0	8 (16) 13 (26) 10 (20) 17 (34) 9 (18) 3 (6) 2 (4) 1 (2) 1 (2)	0.594 0.388 0.333 1.000 1.000 1.000 1.000 1.000 1.000	
*Indication for embolization*				
Rupture with active bleeding Prophylactic Contained bleeding Previous bleeding	5 (71.4) 2 (28.6) 0 0	27 (54) 14 (28) 5 (10) 4 (8)	0.450 1.000 1.000 1.000	
*Type of SAAPs*				
Aneurysm Pseudoaneurysm	5 (71.4) 2 (28.6)	12 (24) 38 (76)	0.020	2.98 [1.51, 5.88]
*Location of SAAPs*				
PDAs Splenic artery/branch Gastroepiploic artery Hepatic artery/branch GDA Left gastric artery/branch Jejunal Right colic	4 (57.1) 1 (14.3) 1 (14.3) 1 (14.3) 0 0 0 0	5 (10) 18 (36) 1 (2) 10 (20) 11 (22) 2 (4) 2 (4) 1 (2)	0.009 0.405 0.232 1.000 0.223 1.000 1.000 0.232	5.71 [1.99–16.33]
Size of SAAPs in mm (mean ± SD)	19.4 ± 16.1	18.4 ± 11.8	0.844	
*Morphology of SAAPs*				
Saccular Fusiform	7 (100) 0	46 (92) 4 (8)	1.000	
Wall calcification	1 (14.3)	3 (6)	0.417	
Wall thrombosis	2 (28.6)	4 (8)	0.153	
*Embolizing materials*				
Coils/microcoils Others*	6 (85.7) 1 (14.3)	41 (82) 9 (18)	1.000	
*Embolized structure*				
SAAP Artery Both artery and SAAP	3 (42.9) 3 (42.9) 1 (14.3)	8 (16) 32 (64) 10 (20)	1.000 0.411 0.333	
Technical success of embolization**	6 (85.7)	45 (90)	0.562	
Immediate complications*** (*n* = 5)	2 (28.6)	3 (6)	0.109	
Non-immediate complications (*n* = 14)	2 (28.6)	12 (24)	1.000	
*Mortality rate*				
30-day 90-day	0 0	7 (14) 12 (24)	0.579 0.325	

All categorical variables are expressed as n (%).

*Polyvinyl alcohol particles, covered stents, vascular plug.

**Considered if the aneurysm was excluded and hemorrhage ceased in cases of rupture.

***Defined by the occurrence during or in the same day of the procedure. CI, confidence interval; DM, diabetes mellitus; HBP, high blood pressure; OR, odds ratio; SAAPs, splanchnic artery aneurysms and pseudoaneurysms; SD, standard deviation; UK, unknown; Y, yes.

The mean size of SAAPs did not differ significantly between patients with and without MALC (19.4 vs. 18.4 mm, *P* = 0.844). All patients with MALC had saccular aneurysms, which was also the most frequent morphology of SAAPs in patients without MALC (92%). Few SAAPs had wall calcification or thrombosis, namely 14.3% and 28.6% of patients with MALC, and 6% and 8% of patients without MALC, respectively.

**Fig. 5:**
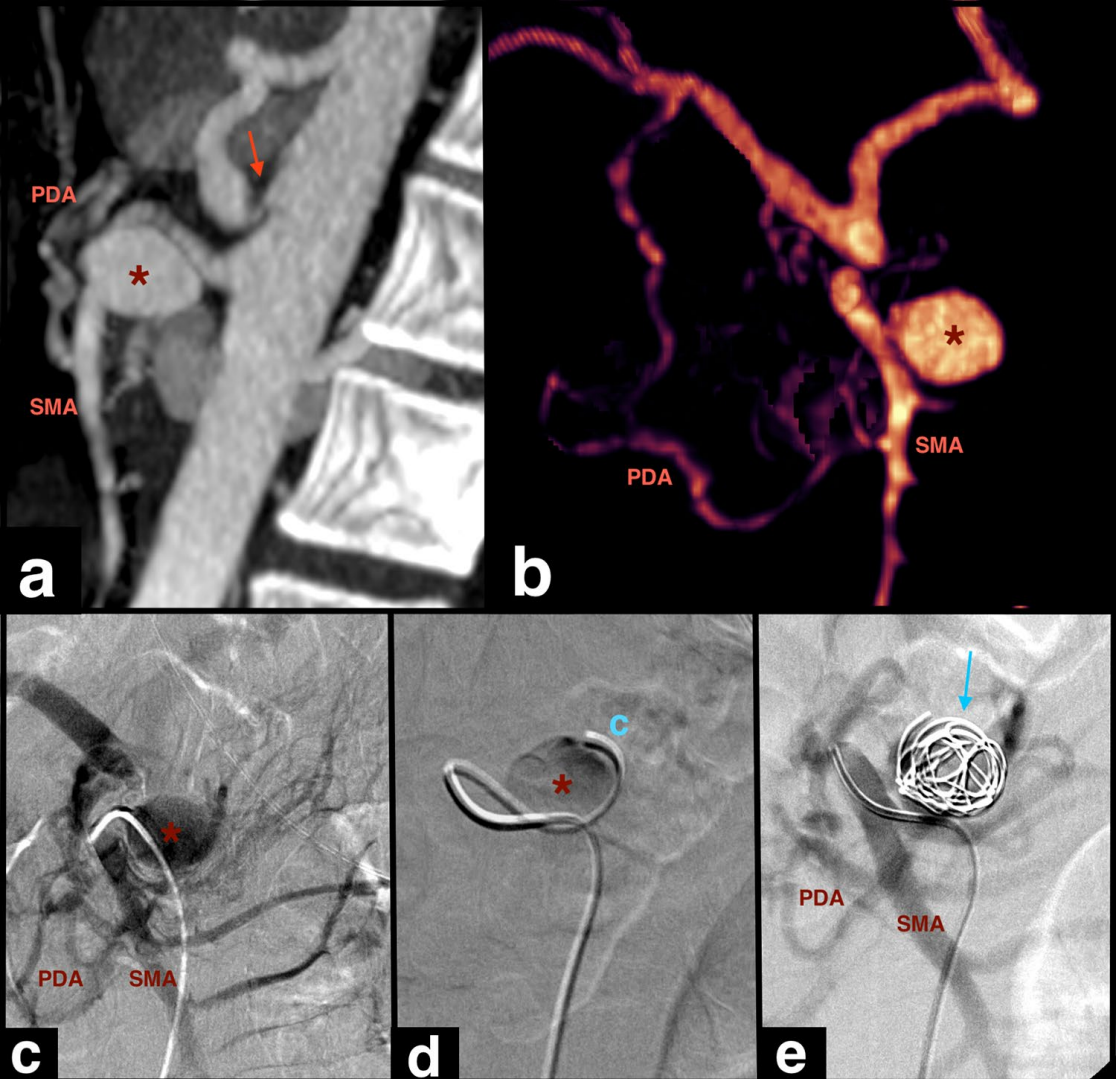
Aneurysm of the inferior pancreaticoduodenal artery in a patient with celiac artery stenosis. Sagittal MIP (**a**) and 3D MIP reconstruction (**b**) CTA images show the celiac artery compression (arrow in A), and the aneurysm (*) measuring 2 cm. Selective superior mesenteric artery (SMA) angiogram (**c**) shows the aneurysm (*). After selective catheterization with a microcatheter (**c** in image **d**), the aneurysm was embolized with coils (arrow in e), with prophylactic intent. CTA, computed tomography angiography; MIP, maximum intensity projection; PDA, pancreaticoduodenal artery

The location of SAAPs in the PDAs (Fig. 5) was more prevalent in patients with MALC (57.1%), whereas it occurred in 10% of patients without MALC. This difference was statistically significant (*P* = 0.009), with a 5.71 higher chance of this location in the first group (95% confidence interval (CI) of [1.99–16.33]). The only other variable that showed a statistically significant association with the presence or absence of MALC was the type of vascular dilatation, with a greater proportion of aneurysms in patients with MALC (71.4%) when compared with those without MALC (24%), whereas pseudoaneurysms were less common (28.6% vs 76%). The risk of aneurysm instead of pseudoaneurysm was 2.98 times higher in patients with MALC (*P* = 0.020, 95% CI [1.51–5.88]).

Coils and microcoils were the most frequent embolization materials used in both patients with and without MALC (85.7% and 82%, respectively). The procedure was successful in most cases, namely in 85.7% and 90% of patients with and without MALC, respectively. Few complications occurred after the procedure, including 5 immediate (in 28.6% and 6% of patients with and without MALC) and 14 non-immediate (in 28.6% and 24% of patients with and without MALC). Specifically, one patient with MALC presenting with a 50 mm splenic artery aneurysm suffered arterial rupture during the procedure and partial permeabilization 3 months later. Other complications in this group included coil embolization (immediate) and recurrent bleeding within 6 months. The mortality rate was 0% in patients with MALC, whereas patients without MALC had 14% and 24% mortality rates at 30 and 90 days, respectively.

**Fig. 6:**
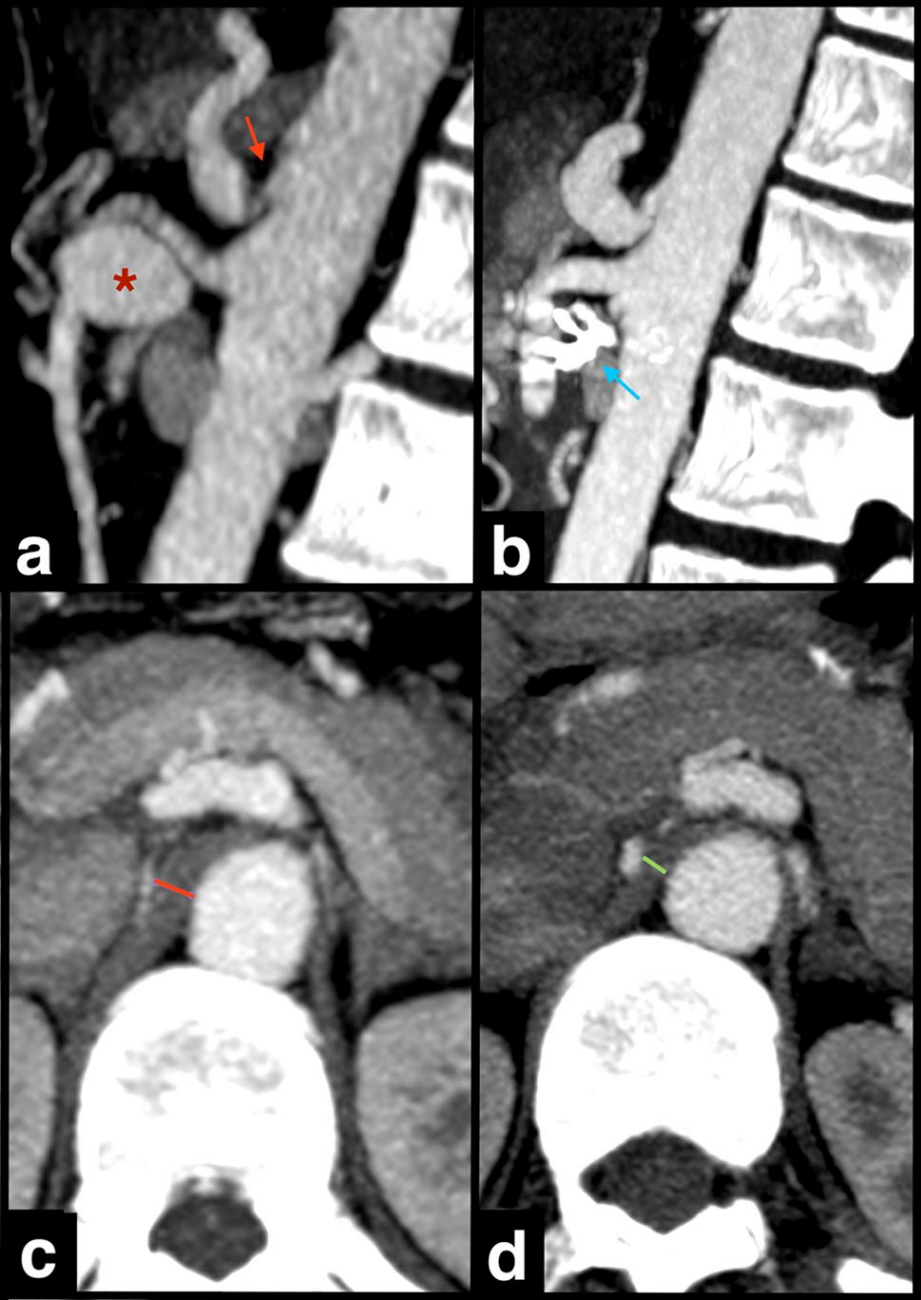
Residual stenosis after surgical incision of the median arcuate ligament (MAL). CTA images from the same patient in Figure, before (**a**, **c**) and 3 months after (**b**, **d**) surgical MAL incision, performed 16 months after the endovascular treatment of the pancreaticoduodenal artery aneurysm (*). Sagittal MIP CTA images (**a**, **b**) show the hook-shaped appearance at the origin of the celiac artery (arrow in A), and the coils within the embolized PDA aneurysm (*). Axial CTA images (**c**, **d**) show reduction of the maximum thickness of MAL, measured in the axial plane, at the level of the CA origin (red and green lines). CTA, computed tomography angiography; MIP, maximum intensity projection

During a mean follow-up of 1967 days (ranging from 401 to 3116 days), only 1 out of 7 patients with MALC performed surgical MAL incision, 16 months after the endovascular treatment of the SA aneurysm. Follow-up CTA at 3 months showed CA residual stenosis, although less severe (Fig. 6). The remainder patients were kept under clinical and imaging surveillance, with no register of aneurysm recurrence or other complications.

Descriptive analysis of cases with CA stenosis caused by MALC and atherosclerosis is presented in Table 2. Patients with atherosclerosis were older (mean age of 76.7 years ± 8.6) than those with MALC (59.7 years ± 18.4, *P* = 0.170). Diabetes mellitus and HBP were frequent in both groups, namely in 42.9% and 71.4% of patients with MALC, and in 100% and 66.7% of patients with atherosclerosis, respectively. Patients with atherosclerotic CA stenosis had pseudoaneurysms caused by gastric ulcer and neoplasm perforation, chronic pancreatitis, and percutaneous transhepatic cholangiography. One patient with MALC had a pseudoaneurysm secondary to biliodigestive surgery. Active bleeding was the greater indication for embolization in both groups (71.4% and 100% of patients with MALC and atherosclerosis, respectively, *P* = 0.1.000). The only exceptions were two cases of prophylactic embolization in the MALC group.

The mean size of SAAPs was larger in patients with MALC (19.4 mm) than in those with atherosclerotic stenosis (12.3 mm, *P* = 0.729). The mean grade of CA stenosis was similar in both groups (70.9% in the MALC group and 68.0% in the atherosclerosis group, *P* = 1.000), although the range was higher in patients with MALC (50% to 100% vs. 62.5% to 71.4%). Median arcuate ligament thickness in patients with MALC ranged from 4 to 10 mm, with a mean of 6.7 mm. In general, the mean caliber of the evaluated splanchnic arteries was greater in patients with MALC, especially in GDA (4.6 vs. 3.3 mm, *P* = 0.059) and PDAs (3.6 vs. 2.1 mm, *P* = 0.153).

The only complication in the atherosclerosis group was recurrent bleeding, solved with a new embolization. The mortality rate at 30 and 90 days in this group was higher (33.3% and 100%, respectively) compared to patients with MALC (0%, *P* = 300 and 0.008).

**Table 2:** Descriptive analysis of cases with CA stenosis caused by median arcuate ligament compression (MALC) and by atherosclerosis

Variables	MALC (*n* = 7)	Atherosclerosis (*n* = 3)	*P-*value
Mean age (years ± SD)	59.7 ± 18.4	76.7 ± 8.6	0.170
*Gender*			
Male Female	4 (57.1) 3 (42.9)	2 (66.7) 1 (33.3)	1.000
*Comorbidities*			
DM HBP	3 (42.9) 5 (71.4)	3 (100) 2 (66.7)	0.200 1.000
Smoking habits (Y/N/UK)	1 (14.3)/ 1 (14.3)/ 5 (71.4)	0/ 0/ 3 (100)	1.000
Alcohol consumption (Y/N/UK)	1 (14.3)/ 1 (14.3)/ 5 (71.4)	1/ 0/ 2 (66.7)	1.000
*Cause of SAAPs*			
MALC Post-biliodigestive surgery Gastric ulcer/neoplasm Chronic pancreatitis Post-PTC	6 (85.7) 1 (14.3) 0 0 0	0 0 1 (33.3) 1 (33.3) 1 (33.3)	0.033 1.000 0.300 0.300 0.300
*Clinical presentation*			
Upper GI bleeding Abdominal pain Anemia Asymptomatic Intra-abdominal hemorrhage Hemobilia Syncope	2 (28.6) 2 (28.6) 1 (14.3) 2 (28.6) 2 (28.6) 0 0	1 (33.3) 1 (33.3) 1 (33.3) 0 0 1 (33.3) 1 (33.3)	1.000 1.000 1.000 1.000 1.000 0.300 0.300
*Indication for embolization*			
Rupture with active bleeding Prophylactic	5 (71.4) 2 (28.6)	3 (100) 0	1.000
*Type*			
Aneurysm Pseudoaneurysm	6 (85.7) 1 (14.3)	0 3 (100)	0.167
*Location of SAAPs*			
PDAs Splenic artery Gastroepiploic artery Hepatic branch GDA	4 (57.1) 1 (14.3) 1 (14.3) 1 (14.3) 0	0 1 (33.3) 0 1 (33.3) 1 (33.3)	0.200 1.000 1.000 1.000 1.000
Mean size of SAAPs in mm ± SD (Min–Max)	19.4 ± 16.0 (7–50)	12.3 ± 4.7 (7–16)	0.729
Morphology of SAAPs Saccular Fusiform	7 (100) 0	3 (100) 0	
Wall calcification	1 (14.3)	0	1.000
Wall thrombosis	2 (28.6)	0	1.000
Mean % of stenosis ± SD (Min–Max)	70.9 ± 18.5 (50–100)	68.0 ± 4.8 (62.5–71.4)	1.000
Mean length of stenosis in mm ± SD (Min–Max)	7.4 ± 3.6 (3–12)	7.3 ± 3.1 (4–10)	
Mean MAL thickness in mm ± SD (Min–Max)	6.7 ± 2.3 (4–10)	-	
*Mean arterial caliber in mm ± SD (Min–Max)*			
SMA GDA PDAs Body/tail of pancreas	8.9 ± 1.4 (6–9) 4.6 ± 1.2 (3.5–7) 3.6 ± 2.1 (2–8) 1.7 ± 0.5 (1–2.5)	7.3 ± 0.6 (7–8) 3.3 ± 0.6 (3–4) 2.1 ± 0.8 (1.4–3) 1.5 ± 0.5 (1–2)	0.412 0.059 0.153 0.555
*Embolizing materials*			
Coils/microcoils Microcoils + PVA Microcoils + covered stent Covered stent	5 (71.4) 1 (14.3) 0 1 (14.3)	2 (66.7) 0 1 (33.3) 0	1.000 1.000 0.300 1.000
Technical success of embolization*	6 (85.7)	3 (100)	1.000
Immediate complications**	2 (28.6)	0	1.000
Non-immediate complications	2 (28.6)	1 (33.3)	1.000
*Mortality rate*			
30-day 90-day	0 0	1 (33.3) 3 (100)	0.300 0.008

All categorical variables are expressed as n (%). *Considered if the aneurysm was excluded and hemorrhage ceased in cases of rupture. **Defined by the occurrence during or in the same day of the procedure. CA, celiac artery; DM, diabetes mellitus; HBP, high blood pressure; GD, gastroduodenal; GDA, gastroduodenal artery; GI, gastrointestinal; MAL, median arcuate ligament; MALC, median arcuate ligament compression; PDAs, pancreaticoduodenal arcades; PTC, percutaneous transhepatic cholangiography; SAAPs, splanchnic artery aneurysms and pseudoaneurysms; SD, standard deviation; UK, unknown; Y, yes

## Discussion

Within a period of 11 years, we found a prevalence of celiac artery compression by MAL in 12.3% (*n* = 7) of patients submitted to endovascular embolization of SAAPs at our institution (*n* = 57). Considering aneurysms only, it was present in 35.2% (greater than the rate reported by Regus et al., although the number of visceral aneurysms in their sample was almost twice the size of ours) [29]. Boll et al. found a much greater prevalence of CA stenosis or occlusion (> 60%) in patients treated with coil embolization, but they only included aneurysms of the GDA or PDA [19]. In their retrospective study of 37 patients with MALS diagnosed at CTA, Heo et al. found SA aneurysms in 24.3% of cases, which were more common in patients with collateral circulation. However, only 2 patients underwent embolization, one due to aneurysm rupture and the other due to complete CA obstruction [5]. These findings suggest that the number of patients with co-existent MALC and SA aneurysms is probably higher than we estimated, given that we only analyzed SAAPs submitted to endovascular treatment, which is still more often performed in the presence of complications.

A high prevalence of MALS has been reported in patients with PDAs aneurysms (33–100%), most probably due to the increased blood flow through these collaterals [1–3, 7, 16]. In our study sample, SAAPs in patients with MALC were also more frequent in PDAs (57.1%, *n* = 4), whereas they occurred in 10% of patients without MALC. This difference was statistically significant (*P* = 0.009), with a 5.71 higher chance of this location in the first group (95% CI [1.99–16.33]). Besides PDAs, some reports have described aneurysms in other locations in patients with MALS [13, 19, 21, 23, 26, 30–37]. In the present sample of patients with MALC, we also found them in the splenic and gastroepiploic arteries.

The only other statistically significant difference we found between patients with and without MALC, was a greater proportion of aneurysms in patients with MALC (71.4%) when compared with those without MALC (24%), whereas pseudoaneurysms were less common (28.6% vs 76%). The risk of aneurysm instead of pseudoaneurysm was 2.98 times higher in patients with MALC (*P* = 0.020, 95% CI [1.51–5.88]). This is an expected finding, as the hypothesized physiopathological explanation for the development of splanchnic artery aneurysms in patients with MALC is the weakening of the wall of small collateral arteries connecting the CA and the SMA beds, due to increased blood flow, hypertension, and shear stress, whereas pseudoaneurysms are usually secondary to wall disruption from other causes [1, 2, 8]. In fact, the only pseudoaneurysm we found in patients with MALC was secondary to biliodigestive surgery, although it is possible that the weakness of the vessel walls induced by MALC may still have been contributory.

Caruana et al.found that most PDAs aneurysms (7 out of 8) were associated with severe CA stenosis, defined as 80–100% stenosis or ≥ 8 mm in length [3]. Bonardelli et al.also reported a preponderance of severe stenosis (56%) in their study sample [38]. In our group of patients with MALC, 50% was the lower value of stenosis, in a patient submitted to prophylactic embolization of a splenic artery aneurysm. In the other cases, the grade of stenosis was higher, ranging from 55 to 100% (mean 70.86%). This may suggest that a greater CA stenosis predisposes to the formation of SA aneurysms. Also, the diameter of collaterals was greater in cases of more severe stenosis. These findings are consistent with those reported by others [39]. However, the grade of CA stenosis may have been underestimated, since CTA scans are usually obtained on inspiration, with partial release of the compression.

In their study of PDAs aneurysms, Antoniak et al.also graded the thickness of MAL into severe or nonsevere, with a threshold value of 4 mm. Most cases (10 out of 15) were severe [37]. Adopting the same grading, all cases in our study were severe, and the mean thickness was 6.3 mm, ranging from 4 to 10 mm. This could also suggest that SAAPs are more prevalent in patients with greater CA stenosis. However, no correlation was found between that measure and the grade of stenosis, but both are influenced by the inspiration status, which was not controlled in this study.

Although some authors reported that most patients with SAAPs and MALC were asymptomatic [37], in our 7 patients with MALC, only two of them were, and rupture was the main indication for embolization (71.4%). This is probably explained by the patient selection, given that we analyzed SAAPs submitted to embolization, which is mostly done following complications, namely rupture. In their review of five SA aneurysms associated with MALS, Sugiyama and Takehara found rupture in 60% of them (2 in PDAs and 1 in epiploic artery), successfully treated with transarterial coil embolization [4]. Boll et al.also found a large proportion of symptomatic patients (85%) in their study of embolized PDA and GDA aneurysms, including 45% ruptured cases [19]. These findings reinforce the potential clinical relevance of SA aneurysms in this group of patients. It is important to be aware of the presence of CA stenosis before the endovascular treatment of PDA aneurysm rupture, as it can influence the vascular approach (CA cannulation may be difficult in some patients) [40]. Also, PDA embolization in patients with CA stenosis can cause ischemia of the upper abdominal organs [1, 40]. Simultaneous blood pressure monitoring of the common hepatic artery has been proposed as a safety measure in patients with MALC, to prevent liver ischemia [41]. We found no reported signs of ischemic damage to the liver or other organs, presumably due to the presence of a rich collateral circulation in all cases.

Interestingly, Tétreau et al.found that ruptured aneurysms due to CA stenosis were significantly smaller than other ruptured aneurysms (9 mm vs. 26 mm) [42]. Although the mean size of ruptured SAAPs in our study was also smaller in cases of MALC (12.9 mm) compared with the others (15.5 mm), we found no statistically significant difference (*P* = 0.538).

Besides CA stenosis secondary to MALC, atherosclerotic disease has also been associated with the presence of PDAs aneurysms. At their article, Bonardelli et al.described 23 patients with concomitant CA stenosis/occlusion and visceral aneurysms, mostly secondary to MALS (44%), followed by atherosclerotic lesions (32%) [38]. Like those authors, we also found that mean age was higher in the atherosclerotic group (76.7 years) compared with MALC group (59.7 years). As expected, the mortality rate was higher in the atherosclerotic group, as these patients have more comorbities and are older.

Management of SA aneurysms remains controversial, given the unpredictability of PDAs aneurysms to rupture, which is neither correlated with the size and multiplicity of the aneurysms, nor the patient’s age [37]. In fact, PDAs aneurysms have a high propensity for spontaneous rupture, regardless of size (as opposed to the other SA aneurysms, in which the risk increases with sizes greater than 2 to 2.5 cm). Also, it as an associated mortality rate of 30% to 50%. Therefore, prophylactic endovascular treatment is recommended to prevent such events [3–5].

However, there is no established consensual approach in terms of when and in whom CA stenosis should be addressed in patients with SA aneurysms and MALC [1, 10, 15, 19–21]. Some authors have recommended CA decompression to prevent organ ischemia and reduce the risk of aneurysm recurrence, either with endovascular procedures (angioplasty and/or stent placement), or surgery (MAL release and/or bypass grafting) (1,2,10,13,15–18]. Splanchnic artery aneurysms and CA stenosis can be addressed simultaneously (one-stage treatment) or separated (two-stage treatment), although there are no established recommendations regarding the optimal order of and duration of time between the two procedures. Also, it depends on the patient’s condition, grade of stenosis, operator’s preference, and availability of a hybrid operation room [15].

Resolution or stability of collateral circulation and aneurysms after treating CA stenosis alone have been reported, presumably due to reduced flow and thrombosis [12, 14, 43–45], but there are reported cases of aneurysm growth after CA decompression [32, 46]. On the other hand, others have obtained good outcomes using aneurysm embolization only [1, 16, 19–27]. However, Takase et al.noted a higher mean age in patients with MALS and PDA aneurysms compared to those without aneurysms (56.9 years vs. 44.6 years), suggesting that longer follow-ups (10 years or longer) may be necessary to detect aneurysm recurrence [27].

In the present study, only 1 out of 7 patients with MALC performed surgical MAL incision 16 months after the endovascular treatment of the SA aneurysm, showing less severe residual CA stenosis in the follow-up CTA 3 months later. The remainder patients had no register of aneurysm recurrence or other complications during a mean follow-up of 1967 days (ranging from 401 to 3116 days). Boll et al.also reported no recurrent PDA or GDA aneurysms after coil embolization alone, although their mean follow-up was shorter (12 months). Still, our study sample is small and further studies with more patients and longer follow-up are necessary to provide evidence for the creation of management recommendations.

### Limitations

The major limitations of our study are its retrospective nature and the relatively small number of patients, inherent to the scarcity of embolized SAAPs. Also, CTA studies weren’t specifically performed for evaluation of CA compression by MAL, so no specification is given in terms of inspiration/inspiration status, precluding homogenization of the analysis. For the same reason, the grade of CA stenosis may have been underestimated, since CTA scans are usually obtained on inspiration, with partial release of the compression. Also, different scanners were used, which may have influenced the arterial measurements, although a good resolution was obtained with CTA protocol in all cases. In addition, we did not evaluate the clinical impact of CA stenosis, namely the presence of symptoms associated with MALC, which we could not accurately access retrospectively in all cases. Finally, the number of cases with different causes of CA stenosis other than MALC was very limited (3 cases of atherosclerotic stenosis), precluding an accurate comparative analysis.

Given all these limitations, to better analyze the prevalence, clinical impact, and outcomes of associated MALC and SAAPs, prospective studies with more patients, well-defined and uniformized imaging studies, and consistent clinical and imaging follow-up and management are required.

## Conclusions

In patients with SAAPs submitted to endovascular embolization, the prevalence of CA compression by MAL is not uncommon. The most frequent location for aneurysms in patients with MALC is in the PDAs. Endovascular management of SAAPs is very effective in patients with MALC, with low complications, even in symptomatic ruptured aneurysms.
